# The unexpected finding of *Parapholidoptera castaneoviridis* in south-eastern Romania (Insecta, Orthoptera, Tettigoniidae)

**DOI:** 10.3897/zookeys.643.10645

**Published:** 2017-01-06

**Authors:** Ionuț Ștefan Iorgu, Dragan Petrov Chobanov, Elena Iulia Iorgu

**Affiliations:** 1 “Grigore Antipa” National Museum of Natural History, Kiseleff Blvd. 1, Bucharest, Romania; 2 Institute of Biodiversity and Ecosystem Research, Bulgarian Academy of Sciences, 1 Tsar Osvoboditel Blvd., 1000 Sofia, Bulgaria

**Keywords:** Balkan Peninsula, bush-cricket, calling song, relict population, distribution

## Abstract

The Balkano-Anatolian genus *Parapholidoptera* comprises 21 species and the westernmost one, *Parapholidoptera castaneoviridis*, previously recognized to occur in western Turkey, north-eastern Greece and south-eastern Bulgaria is recorded for the first time from south-eastern Romania, almost 300 km away from the closest known locality. Illustrations and measurements of morphological characters are given and the male calling song from this new, northernmost population is described.

## Introduction

With 21 described species, the genus *Parapholidoptera* Mařan is distributed from the Caucasus in the east, to the Balkans in the west, across the Anatolian Plateau and reaching Israel and Jordan in the south (Çiplak 2000, Katbeh Bader and Massa 2001, Çiplak 2004, Eades et al. 2016). Within the genus, Çiplak (2000) defines two clades: *Parapholidoptera castaneoviridis* group with a south-western distribution and *Parapholidoptera distincta* group, occurring in north-eastern Anatolia and the Caucasus. It seems that the Anatolian mountain ranges played an important role in the speciation and distribution of the genus (Çiplak 2004), 16 species being endemic to various localities and mountains in Turkey, Georgia, Armenia and Jordan.

*Parapholidoptera castaneoviridis* (Brunner von Wattenwyl, 1882) is one of the most widely distributed species of this genus, known to occur from the north Aegean region of Turkey, Samothrace Island, Macedonia and Thrace regions in Greece, Strandzha Mountains and the territory eastwards from Eastern Rhodopes in Bulgaria (Ingrisch and Pavićević 1985, Çiplak 2000, Popov and Chobanov 2004). The species is characterized by a cylindrical pronotum, extended metazona, male with last tergum light-colored, with two small, straight processes and females with a long, straight ovipositor and subgenital plate narrowly rounded, with a deep groove (Çiplak 2000).

The finding of *Parapholidoptera castaneoviridis* in Romania, the northernmost known location in the distribution of this species, is discussed.

## Material and methods

The first specimens of *Parapholidoptera castaneoviridis* were found while actively searching for orthopterans in the forest clearings near Ciucurova village (Tulcea county, south-eastern Romania), in the summer of 2016. The bush-crickets were collected at daytime, in xerophytic *Quercus* forest clearings with scrub, at altitudes of approximately 200 m ASL. Individuals were identified according to genital morphology and calling song oscillographic structure. Photos were taken with a Canon EOS 6D DSLR camera and a Canon EF 180 mm f3.5 macro lens. Acoustic recordings were made at night, in laboratory conditions with an Edirol R-09HR digital recorder (sampling rate 96 kHz, 24-bit amplitude resolution, microphone response frequency up to 45 kHz) and a Knowles electret condenser microphone connected to a PC through a TransitUSB external sound card (48 kHz, 16-bit) and the sound analysis was run with Audacity 2.1.2 and Batsound 4 software. As song element durations are usually temperature dependent, the ambient air temperature was measured during these recordings.

**Figure 1.**
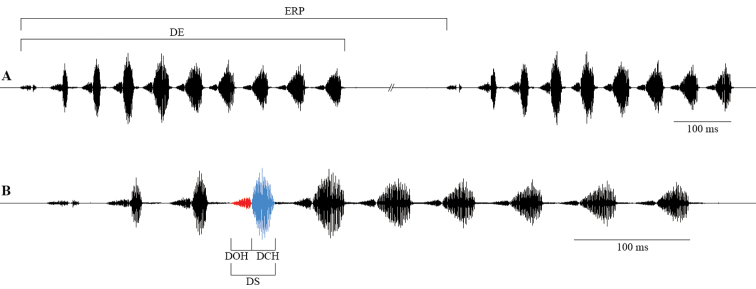
Studied male calling song oscillographic characters in *Parapholidoptera castaneoviridis*: **A** sequence of two echemes **B** detailed echeme (red – opening hemisyllable, blue – closing hemisyllable). Abbreviations: **ERP** – echeme repetition period; **DE** – duration of an echeme; **DOH** – duration of an opening hemisyllable; **DCH** – duration of a closing hemisyllable; **DS** – duration of a syllable.

The bioacoustic terminology is adopted mainly from Ragge and Reynolds (1998): *calling song* – song produced by an isolated male; *syllable* – the song produced by one to-and-fro movement of the tegmina; *hemisyllable* – the song produced by one unidirectional movement of the tegmina (opening or closing); *echeme* – a first order assemblage of syllables; *echeme sequence* – a first order assemblage of echemes. The following oscillographic characters were measured in the songs of three males from Ciucurova and compared to previously known results (Heller 1988, Sevgili et al. 2011): *duration of an echeme* (DE) – the time elapsed from the beginning of first syllable to the end of last syllable of an echeme; *duration of a syllable* (DS) – the time elapsed from the beginning of first impulse to the end of last impulse of a syllable; *duration of an opening hemisyllable* (DOH) – the time elapsed from the beginning of first impulse to the end of last impulse of an opening hemisyllable; *duration of a closing hemisyllable* (DCH) – the time elapsed from the beginning of first impulse to the end of last impulse of a closing hemisyllable; *echeme repetition period* (ERP) – the time elapsed from the beginning of an echeme to the beginning of the next echeme (Fig. 1); *echeme recurrence rate* – density of echemes repetition in a specific time period.

**Figure 2.**
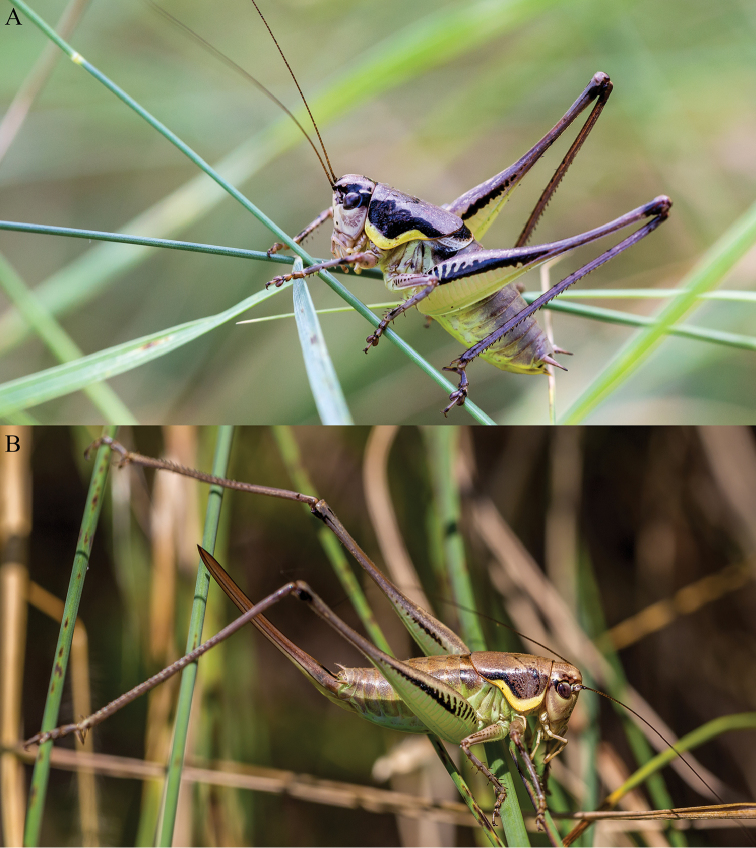
The habitus of *Parapholidoptera castaneoviridis* in the northernmost known location: **A** male **B** female (Romania, Ciucurova, 2016.07.03).

*Examined material*: 2 ♂♂ 2 ♀♀, 2016.07.03, forest clearing north of Ciucurova village, Tulcea county, Romania, 44.9576°N 28.5245°E, 190 m ASL (leg. I. Ș. Iorgu); 1 ♂ 2 ♀♀, 2016.07.30, same locality (leg. I. Ș. Iorgu & E. I. Iorgu) (Fig. 2A, B).

**Figure 3.**
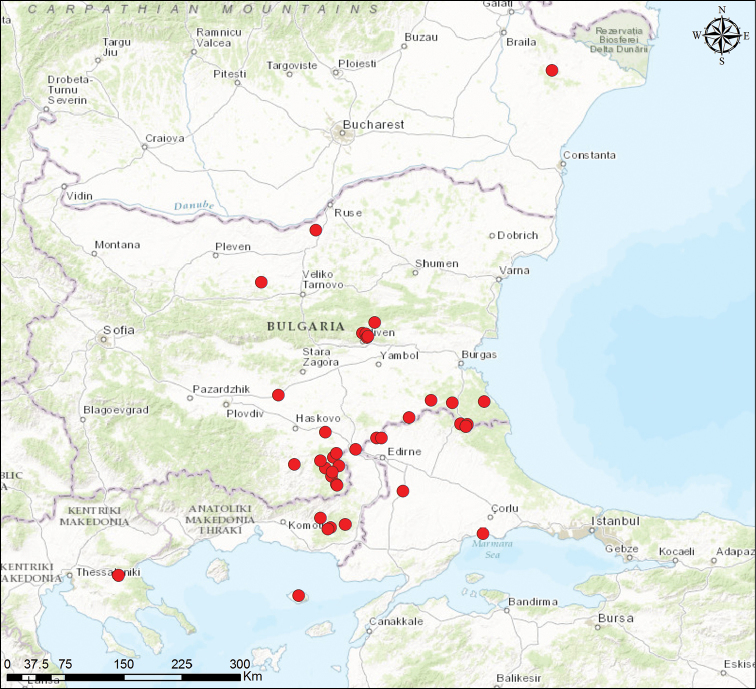
Geographic distribution of *Parapholidoptera castaneoviridis* in the Balkan Peninsula (for a complete list of localities see the Suppl. material 1).

*Other material recorded in the Balkan Peninsula*: see Suppl. material 1 and Figure 3.

*Acoustic recordings*: 2 ♂♂, 2016.07.03, Ciucurova, Romania (temperature 26°C); 1 ♂, 2016.07.30, same locality (28°C); 1 ♂, 2007.06.26, Strandzha Mountains, Malko Turnovo - Gradishteto hill, Bulgaria, 41.965°N 27.491°E, 650 m ASL, temperature 25°C (leg. D. P. Chobanov & M. Ilieva).

### Calling song

**Figure 4.**
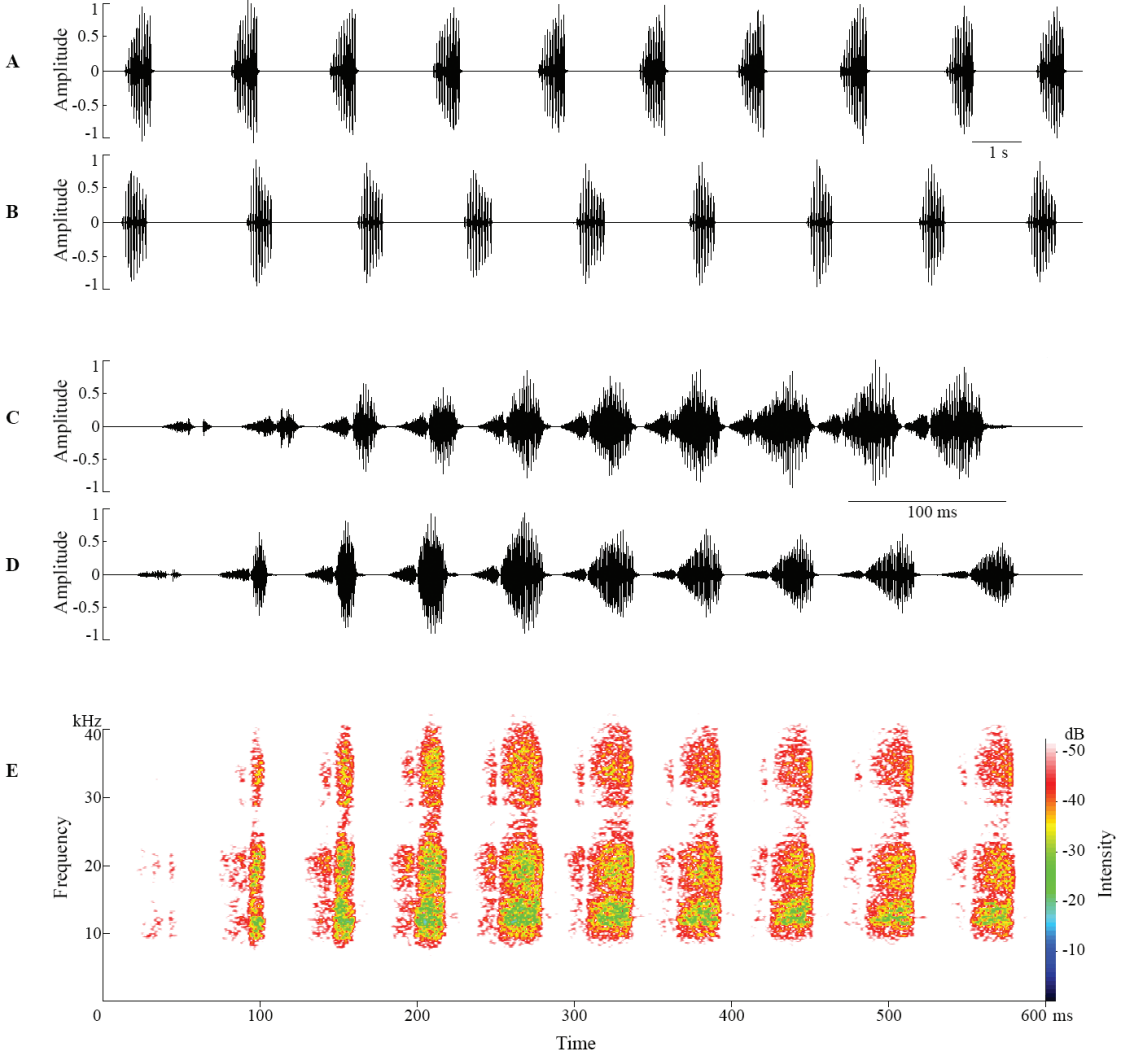
*Parapholidoptera castaneoviridis* male calling song: **A, B** oscillographic representation of an echeme sequence **C, D** detailed echeme **E** spectrogram (referring to the detailed echeme **D**). **A, C** Bulgaria, Strandzha Mts., Malko Turnovo, 25°C **B, D, E** Romania, Ciucurova, 26°C.

**Figure 5.**
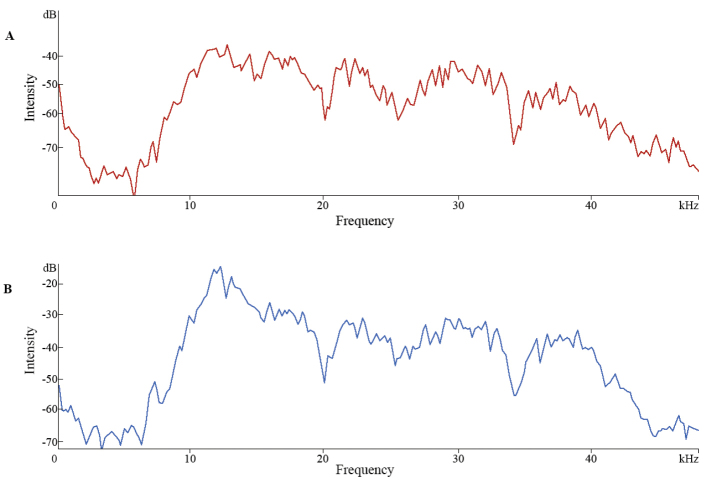
*Parapholidoptera castaneoviridis* male calling song spectrum: **A** opening hemisyllable frequencies (red) **B** closing hemisyllable frequencies (blue). Function: Hanning window; size: 512 (Romania, Ciucurova, 26°C).

Typically, the males produce their calling song in the evening and at night. In the recorded males, the calling song consists of a long series of echemes, each echeme lasting for 426–583 ms (mean ± SD 503.87 ± 46.81 ms) and containing 8–10 syllables (mean ± SD 8.49 ± 0.52) (Fig. 4). The recurrence rate is fairly 20–30 / minute, the echeme repetition period being 2122–2760 ms (mean ± SD 2408.28 ± 197.71 ms). Syllables are quite short (39–54 ms, mean ± SD 47.28 ± 4.1 ms) and consist of two distinct parts: a shorter, lower amplitude opening hemisyllable (15–20 ms, mean ± SD 17.55 ± 1.64 ms) and a longer, higher amplitude closing hemisyllable (20–39 ms, mean ± SD 29.73 ± 4.49 ms). Acoustic signal amplitude modulation pattern is increasing and decreasing in both the opening and closing hemisyllables. The detailed descriptive statistics of the song characters are presented in Table 1. The calling song has the dominant frequency components between 7 kHz and up to more than 45 kHz (45 kHz being the upper limit of microphone frequency response in our recordings), with a main peak at about 12 kHz in both opening and closing hemisyllables (Fig. 5).

**Table 1.** Descriptive statistics of male calling song parameters in *Parapholidoptera castaneoviridis* from south-eastern Romania (n = 100 measurements/male from 3 males). All values are given in milliseconds. Abbreviations: **DE** – duration of an echeme; **DS** – duration of a syllable; **DOH** – duration of an opening hemisyllable; **DCH** – duration of a closing hemisyllable; **ERP** – echeme repetition period.

	DE	DS	DOH	DCH	ERP	Number of syllables / echeme
Max.	583	54	20	39	2760	10
Min.	426	39	15	20	2122	8
Mean	503.87	47.28	17.55	29.73	2408.28	8.49
SD	46.816	4.102	1.641	4.49	197.719	0.522

## Discussion

Although recently *Parapholidoptera* was divided in two groups relying exclusively on morphological traits (Çiplak 2000), the genus is characterized by a more or less uniform calling song, especially among the *Parapholidoptera castaneoviridis* group – multi-syllabic echemes separated by silent intervals lasting for several seconds, with differences in the syllable number per echeme (Heller 2006).

**Figure 6.**
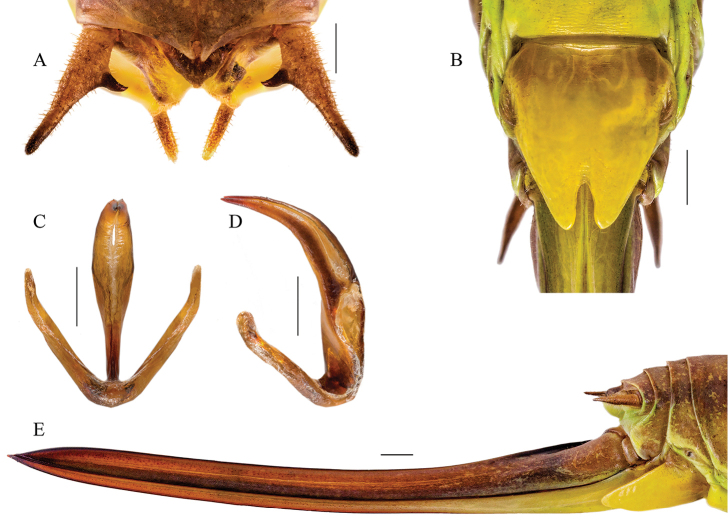
The eidonomy of *Parapholidoptera castaneoviridis* specimens from south-eastern Romania: **A** male cerci **B** female subgenital plate **C, D** titillator **E** ovipositor. Scale bar 1 mm.

The males discovered in Romania are characterized by a homogeneous song pattern with the specimens from Strandzha Mountains and Turkey: variable echemes consisting of 5–8 (Sevgili et al. 2011), 10–12 or 11–13 syllables (Heller 1988, 2006), lasting for 268–420 ms (Sevgili et al. 2011) or more than 500 ms (see oscillogram in Heller 1988) and repeated at 1.5–3 s (Sevgili et al. 2011) or up to 2.78–4.20 s (Heller 1988) are produced by individuals from the core area of species distribution (south-eastern Balkans and western Asia Minor). Having 8–10 syllables/echeme and lasting for 426–583 ms, with a repetition period of 2.1–2.7 s, the song of Romanian specimens fits well within this description. Moreover, the morphological structures and measurements of specimens from the newly found population (Fig. 6) are very similar with the ones described by Çiplak (2000) (for a complete numeric comparison, see Table 2).

**Table 2.** Morphological measurements in *Parapholidoptera castaneoviridis* from south-eastern Romania (n = 3♂♂ 4♀♀), compared with Turkey and Greece (data from Çiplak 2000). All values are given in millimetres.

		Vertex	Scapus	Pronotum	Body	Hind femur	Ovipositor
Romania	♂	2–2.3 (mean 2.16±0.15)	0.9–1 (mean 1±0.1)	7.7–8.7 (mean 8.2±0.5)	23–25 (mean 24±1)	23–24 (mean 24±1)	–
	♀	2–2.3 (mean 2.2±0.1)	0.9–1 (mean 1±0.1)	8.4–8.8 (mean 8.6±0.2)	22–25 (mean 23±1)	26.5–27.5 (mean 27.1±0.42)	21–22 (mean 22±1)
Turkey, Greece	♂	1.8–2.2	0.9–1	8.4–10.8	19–26	21–24.5	–
	♀	2.2–2.3	0.9–1.1	8.3–10.5	20–27	24–28	19–26

*Parapholidoptera castaneoviridis* is a micropterous insect with limited dispersal abilities. The newly discovered population from the northern area of Dobrogea most likely persisted as relict from a previously wider distribution when the forest habitat was continuous, rather than being the result of a recent expansion. This hypothesis may be supported by the isolated former findings of this species in northern Bulgaria, while recently it has not been found in this area, regardless of the significant collecting efforts that have been made in the northeastern part of this country. Currently, the species is common in south-eastern Bulgaria (D. Chobanov, pers. obs.; fig. 3), where it can be found both in natural and agricultural areas, mostly tolerating dry warm microclimate in scrub-grass associations or thermophilic sparse forests.

Numerous recent studies targeting the Balkan orthopteran fauna confirmed the need of exploring unknown territories for obtaining a complete picture of the species geographic ranges (eg. Çiplak et al. 2007, Chobanov and Heller 2010, Chobanov et al. 2013, Chobanov 2014, Kaya et al. 2015, Iorgu et al. 2016). The finding of the only Balkan *Parapholidoptera* species in Romania is just another example of how exhaustive field work in a previously poorly studied area could produce unexpected results, providing a better understanding of the currently interrupted distribution area of a formerly wider spread species.

## Supplementary materials

Supplementary material 1 Occurrence records of *Parapholidoptera castaneoviridis* in the Balkan Peninsula Data type: occurrence Explanation note: The occurrence records of *Parapholidoptera castaneoviridis* in the Balkan Peninsula, from literature and collected by the authors. This dataset is made available under the Open Database License (http://opendatacommons.org/licenses/odbl/1.0/). The Open Database License (ODbL) is a license agreement intended to allow users to freely share, modify, and use this Dataset while maintaining this same freedom for others, provided that the original source and author(s) are credited.Ionuț Ștefan Iorgu, Dragan Petrov Chobanov, Elena Iulia Iorgu
